# Inhibition of Tryptophan Hydroxylases and Monoamine Oxidase-A by the Proton Pump Inhibitor, Omeprazole—*In Vitro* and *In Vivo* Investigations

**DOI:** 10.3389/fphar.2020.593416

**Published:** 2020-11-26

**Authors:** Nibal Betari, Kristoffer Sahlholm, Xavier Morató, Héctor Godoy-Marín, Olga Jáuregui, Knut Teigen, Francisco Ciruela, Jan Haavik

**Affiliations:** ^1^ Department of Biomedicine, University of Bergen, Bergen, Norway; ^2^ Department of Integrative Medical Biology, Wallenberg Centre for Molecular Medicine, Umeå University, Umeå, Sweden; ^3^ Department of Neuroscience, Karolinska Institutet, Stockholm, Sweden; ^4^ Pharmacology Unit, Department of Pathology and Experimental Therapeutics, Faculty of Medicine and Health Sciences, Institute of Neurosciences, University of Barcelona, L’Hospitalet de Llobregat, Barcelona, Spain; ^5^ Neuropharmacology and Pain Group, Neuroscience Program, Institut d’Investigació Biomèdica de Bellvitge, IDIBELL, L’Hospitalet de Llobregat, Barcelona, Spain; ^6^ Department of Clinical Neuroscience, Karolinska Institutet, Stockholm, Sweden; ^7^ Scientific and Technological Centers of University of Barcelona (CCiTUB), Barcelona, Spain; ^8^ Division of Psychiatry, Bergen Center of Brain Plasticity, Haukeland University Hospital, Bergen, Norway

**Keywords:** serotonin, drug repurposing, high-throughput screening assay, molecular docking analysis, enzyme assay, allosteric site

## Abstract

Serotonin (5-HT) is a hormone and neurotransmitter that modulates neural activity as well as a wide range of other physiological processes including cardiovascular function, bowel motility, and platelet aggregation. 5-HT synthesis is catalyzed by tryptophan hydroxylase (TPH) which exists as two distinct isoforms; TPH1 and TPH2, which are responsible for peripheral and central 5-HT, respectively. Due to the implication of 5-HT in a number of pathologies, including depression, anxiety, autism, sexual dysfunction, irritable bowel syndrome, inflammatory bowel disease, and carcinoid syndrome, there has been a growing interest in finding modulators of these enzymes in recent years. We thus performed high-throughput screening (HTS) using a fluorescence-based thermal shift assay (DSF) to search the Prestwick Chemical Library containing 1,280 compounds, mostly FDA-approved drugs, for TPH1 binders. We here report the identification of omeprazole, a proton pump inhibitor, as an inhibitor of TPH1 and TPH2 with low micromolar potency and high selectivity over the other aromatic amino acid hydroxylases. The S-enantiomer of omeprazole, esomeprazole, has recently also been described as an inhibitor of monoamine oxidase-A (MAO-A), the main enzyme responsible for 5-HT degradation, albeit with lower potency compared to the effect on TPH1 and TPH2. In order to investigate the net effect of simultaneous inhibition of TPH and MAO-A *in vivo*, we administered high-dose (100 mg/kg) omeprazole to CD-1 mice for 4 days, after which the animals were subjected to the tail suspension test. Finally, central (whole brain) and peripheral (serum) 5-HT content was measured using liquid chromatography-mass spectrometry (LC-MS). Omeprazole treatment significantly increased 5-HT concentrations, both in brain and in serum, and reduced the time spent immobile in the tail suspension test relative to vehicle control. Thus, the MAO-A inhibition afforded by high-dose omeprazole appears to overcome the opposing effect on 5-HT produced by inhibition of TPH1 and TPH2. Further modification of proton pump inhibitor scaffolds may yield more selective modulators of 5-HT metabolism.

## Introduction

Serotonin (5-hydroxytryptamine, 5-HT) is a monoamine neurotransmitter, synthesized mainly in the enterochromaffin cells of the gut and the raphe nuclei of the central nervous system (CNS). 5-HT is implicated in the control of a wide variety of physiological functions in the CNS and the peripheral nervous system (PNS), such as sleep, pain, gut motility, appetite, sexual behavior and mood, insulin secretion, and heart and brain development (Cote et al., 2007; Berger et al., 2009). The key enzymes that promote the biosynthesis and degradation of 5-HT are the tryptophan hydroxylases (TPHs) (McKinney et al., 2005) and monoamine oxidase (MAO) (Tong et al., 2013), respectively.

The TPHs catalyze the first and rate limiting step of the biosynthesis of 5-HT starting from the amino acid, tryptophan and are encoded by two different genes; TPH1, which is responsible for the synthesis of 5-HT in peripheral tissues and the pineal gland, and TPH2, which is the main source of 5-HT in CNS tissues (Walther and Bader, 2003; Walther et al., 2003). Monoamine oxidases (MAOs) are flavin adenine dinucleotide (FAD) co-factor-dependent enzymes involved in the regulation of neurotransmitter levels in both peripheral and central tissues (Slotkin, 1999; De Colibus et al., 2005). The MAOs exist as two distinct isoforms, MAO-A and MAO-B, which share highly conserved features, but are expressed in different cell types and exhibit different regulatory properties and substrate specificities (Zhang et al., 2019). The MAO-A isoform controls 5-HT catabolism by catalyzing its oxidation to 5-hydroxyindoleacetic acid and is important for maintaining physiological 5-HT levels in various tissues (Tong et al., 2013).

Both TPHs and MAO-A are thus attractive targets for treatment of disorders linked to the dysregulation of 5-HT levels either in the CNS or PNS. Several TPH1 inhibitors have been developed for treating diseases associated with elevated 5-HT levels in peripheral tissues such as carcinoid syndrome, irritable bowel disease with diarrhea, inflammatory bowel disease, pulmonary arterial hypertension, obesity, and diabetes (Manocha and Khan, 2012; Margolis et al., 2014; Kim et al., 2015; Waloen et al., 2017; Matthes and Bader, 2018); e.g., *p*-chlorophenylalanine (fenclonine, pCPA) (Engelman et al., 1967), which was the first TPH inhibitor to reach clinical studies. However, due to the occurrence of depression as a side effect, the clinical development of this compound was discontinued (Zimmer et al., 2002). TPH inhibitor-induced depression is likely a result of TPH2 inhibition and consequent reduction of central 5-HT. Hence, the development of TPH1 inhibitors which cannot cross the blood–brain barrier remains an appealing challenge to medicinal chemists. With a peripheral action in mind, derivatives of pCPA, e.g.; LP-521834, LP-534193, and LP-533401, have been developed as TPH1 inhibitors by Lexicon Pharmaceuticals (Cianchetta et al., 2010), whereas other compounds including KAR5585 and KAR5417, have been synthesized by Karos Pharmaceuticals for the same target (Goldberg et al., 2017). Few of these compounds have reached clinical studies; as of today, only telotristat ethyl (LX-1032), developed by Lexicon, has received FDA approval for treatment of carcinoid syndrome (Markham, 2017). The two other members of the aromatic amino acid hydroxylase family, tyrosine hydroxylase (TH) and phenylalanine hydroxylase (PAH), share a highly conserved active site with TPH1 and TPH2 (Teigen et al., 2007). Novel TPH1 inhibitors with improved selectivity over tyrosine hydroxylase (TH) and phenylalanine hydroxylase (PAH) would therefore be desirable in order to reduce undesired effects on the metabolism of other monoamines.

Increased MAO-A activity has been associated with depression (Meyer et al., 2006) and several FDA-approved MAO-A inhibitors have been used for the treatment of depression and anxiety (Finberg and Rabey, 2016). Among these inhibitors, iproniazid was first described in 1983 by Lehmann and Kline for treating depression (Sandler, 1990). Likewise, several non-subtype-selective, irreversible MAO inhibitors were developed from this compound; e.g., tranylcypromine, which is used clinically as an antidepressant despite causing potentially serious “cheese effect” adverse reactions, precipitating episodes of high blood pressure upon intake of foods rich in tyramine, including some aged cheeses (Gillman, 2011). Another serious and sometimes lethal adverse effect documented among patients taking MAO inhibitors is serotonin syndrome, typically including neuromuscular and autonomic hyperactivity (such as tremor and fever) and agitation. Serotonin syndrome may be triggered when combining MAO inhibitors with serotonergic drugs such as selective serotonin reuptake inhibitors (SSRIs) (Gillman, 2006).

Therapeutic targeting of 5-HT dysregulation remains a challenge in drug discovery, since 5-HT signaling is involved in a wide spectrum of biological functions in the human body. Safety and selectivity are thus important issues to be considered before the drug candidate reaches clinical trials. Attempts at repositioning of already FDA-approved drugs for novel indications have increased rapidly in recent years. The potential value of this strategy lies in the reduction of the time and economic risk typically associated with bringing new drug candidates to clinical trials (Keiser et al., 2009; Corsello et al., 2017). Furthermore, screening approved drugs for unknown off-targets may reveal valuable information regarding adverse reactions associated with their use (Keiser et al., 2009). Here, we used a high-throughput screening (HTS) *in vitro* assay based on differential scanning fluorimetry (DSF) in order to find TPH1 binders among the mostly (95%) FDA-approved compounds of the Prestwick Chemical Library (1,280 substances).

Results showed that the best-selling, over-the-counter proton pump (H^+^/K^+^-ATPase) inhibitor, omeprazole, inhibited TPH1 and TPH2 in the low micromolar range and displayed excellent selectivity over the other closely related aromatic amino acid hydroxylases, i.e.; TH and PAH. Proton pump inhibitors are used clinically for the treatment of gastroesophageal reflux disease and peptic ulcer disease (Herszenyi et al., 2020). These compounds are prodrugs which are absorbed from the gastro-intestinal tract and converted into their active sulfonamide form, which irreversibly binds and inhibits H^+^/K^+^-ATPase, in the acidic canaliculi of the gastric parietal cells (Howden, 1991). Proton pump inhibitors are sold without prescription in many countries and are considered among the safest classes of drugs, generally being well tolerated. Interestingly, the S-enantiomer of omeprazole; esomeprazole, was recently reported to inhibit MAO-A (Petzer et al., 2013). In order to gain more insight into the serotonergic profiles of proton pump inhibitors, we characterized the inhibitory effects of omeprazole, esomeprazole, and their commercially available analogues on TPH1 and TPH2. The activities of omeprazole and its analogues at MAO-A were also assessed. Finally, the effect of omeprazole on *in vivo* 5-HT levels in brain and serum, as well as behavior, was evaluated.

## Materials and Methods

### Materials

The Prestwick Chemical Library was purchased from Prestwick Chemical labs (Paris, France). SYPRO Orange, reagents and compounds were purchased from Sigma-Aldrich (St. Louis, MO, USA) with a purity of at least 95%. Chromatography materials for enzyme purification and enzymatic activity assay were purchased from Amersham Biosciences, GE Healthcare (Chicago, IL) 6R-L-erythro-5,6,7,8-tetrahydrobiopterin (BH_4_) was purchased from Schircks Laboratories (Bauma, Switzerland).

The ΔNH102-ΔCOOH402 human TPH1 gene was cloned into the pET23a vector (six-His C-terminal fusion; Merck KGaA, Darmstadt, Germany) between the NdeI and XhoI restriction sites, and expressed and purified as described previously (Wang et al., 2002; Betari et al., 2020). Truncated TPH1 was used since the full-length enzyme tends to aggregate in bacteria and yeast extracts and during purification (Wang et al., 2002; McKinney et al., 2005), resulting in a much lower protein yield compared to the truncated version. Enzymatic activity of truncated TPH1 is equal to that of the full-length protein (Betari et al., 2020). For comparison, full-length TPH1 was used as a control in a subset of experiments with omeprazole.

Full-length WT TPH1 and TPH2 were expressed as N-terminal six-His-maltose-binding protein fusion proteins in pETM-41 and were cleaved and purified as described previously (McKinney et al., 2004; Winge et al., 2007). Human WT PAH and TH were expressed and isolated as described previously (Flydal et al., 2012; Bezem et al., 2016).

### Differential Scanning Fluorimetry Assay

Human doubly truncated TPH1 (ΔNH102-ΔCOOH402) was used for HTS using the DSF method (a fluorescence-based thermal stability assay) (Lo et al., 2004; Niesen et al., 2007). The protein was overexpressed, purified and stored in 20 mM Na HEPES (sodium salt of HEPES, pH 7.0), 200 mM NaCl as previously described (Wang et al., 2002; Betari et al., 2020).

The Prestwick Chemical Library, which consists of 1,280 small molecules (95% FDA-approved drugs), was screened using the DSF method. The compound stocks were prepared at a concentration of 10 mM in DMSO. SYPRO Orange was utilized at 1,000x dilution to monitor protein unfolding using a Lightcycler 480 Real-Time PCR System (Roche Applied Science, Penzberg, Germany), using the 384 well format. The enzyme was diluted in 20 mM Na HEPES, 200 mM NaCl (pH 7.0) buffer to a final concentration of 0.075 mg/ml and compounds were added to a final concentration of 200 μM. Control experiments with 2% DMSO were performed in the absence of ligand on each 384-well plate. Next, samples were incubated at room temperature for 30 min before measurements were started on the Lightcycler^®^ 480 Real-Time PCR System. The thermal shift curves were recorded in the presence and absence of the compounds from 20°C to 95°C with four acquisitions per °C, including a 10 s hold at 20°C before and after the experiment. Part of the results of the DSF screen have been presented previously.

### Tryptophan HydroxylaseEnzymatic Activity Assay

Enzymatic activity assays were performed with selected hits from the DSF screen in the presence or absence of 100 µM of tested compound. Control experiments were performed in the presence of 1% DMSO in the absence of ligand. TPH1 activities were assayed at 37°C in a standard reaction mixture (100 µL final volume) containing 40 mM Na HEPES, 0.05 mg/ml catalase, 10 µM ferrous ammonium sulfate, and 20 μM l-tryptophan (L-Trp). The enzymatic reaction was initiated by adding 200 μM BH_4_ and 2 mM DTT (final concentrations) and stopped by precipitation with 2% (v/v) acetic acid in ethanol. 5-hydroxy tryptophan (5-OH-Trp) was quantified using High Performance Liquid Chromatography (HPLC) essentially as described previously with minor modifications (McKinney et al., 2004; Winge et al., 2007; Betari et al., 2020). Compounds inhibiting TPH1 activity by more than 50% in the preliminary activity assay were selected for further dose-response analyses and IC_50_ determination. The effect of omeprazole and its analogues were determined on both the doubly truncated TPH1 protein and WT full-length TPH2 protein. The effect of omeprazole was also studied at full-length WT TPH1.

### Mechanism of Action and Kinetics Analysis of Omeprazole

The mode of TPH1 inhibition by omeprazole was investigated using doubly truncated TPH1 (ΔNH102-ΔCOOH402) as described previously (Betari et al., 2020). The TPH1 activity assay was performed as indicated above in the absence and presence of omeprazole (1, 10, or 100 µM). 5-OH-Trp formation was measured either at a fixed concentration (200 µM) of the cofactor, BH_4_, and varying concentrations (0.625–20 µM) of the substrate, L-Trp, or in the presence of varying concentrations of BH_4_ (2.4–200 µM) and a fixed concentration (20 µM) of L-Trp. Kinetic parameters were estimated by fitting the Michaelis-Menten model to the data using nonlinear regression.

### Selectivity Studies of Omeprazole – Enzymatic Activity Assays Using Other Aromatic Amino Acid Hydroxylases

The selectivity of omeprazole for inhibiting TPH1 over the other aromatic amino acid hydroxylases; TPH2, TH, and PAH, was investigated using *in vitro* functional enzyme assays. Purified WT human TPH2, PAH, and TH were used for this purpose, and the activity assays for each enzyme was performed as described previously (Haavik and Flatmark, 1980; Winge et al., 2007; Aubi et al., 2015; Bezem et al., 2016).

### Monoamine Oxidase-A Enzymatic Activity Assay

The effects of omeprazole and its analogues on MAO-A activity were determined using a commercial MAO-A Inhibitor Screening Kit. The experiments were conducted following the manufacturer’s (BioVision, Inc, Milpitas, CA) instructions and were based on the fluorimetric detection of H_2_O_2_, a byproduct of the enzymatic activity of MAO-A, by measuring the fluorescence (Excitation/Emission at 535/587 nm) kinetically at 25°C for 15 min using a Tecan Spark plate reader (Tecan Group Ltd, Männedorf, Switzerland).

### Molecular Docking

Molecular docking was performed with Glide which is part of the Schrodinger program package (**Schrödinger release 2020–1**: Glide, Schrödinger, LLC, New York, NY, 2020). The “Induced Fit Docking” (IFD) protocol (**Schrödinger release 2020–1**: Induced Fit Docking protocol; Glide, Schrödinger, LLC, New York, NY, 2016; Prime, Schrödinger, LLC, New York, NY, 2020) was used to flexibly dock omeprazole into MAO-A and TPH1. Four forms of omeprazole were prepared for docking, i.e., the S and R enantiomers as well as protonated and neutral forms. Coordinates used for docking were those of human MAO-A in complex with harmine (PDB identification code 2Z5X (Son et al., 2008); and human TPH1 in complex with its biopterin cofactor (PDB identification code 1MLW) (Wang et al., 2002). Sidechains of protein pocket-residues were re-oriented to accommodate omeprazole and to optimize calculated interaction energies. The cofactor, harmine as well as the water molecules were removed from the two protein structures prior to docking. Harmine defined the center of the pocket where omeprazole was docked into MAO-A. Two binding pockets were defined for docking to TPH1; 1) the cofactor pocket, defined by the center of the cofactor in the crystal structure, and 2) the postulated allosteric pocket, where the center of the docking grid box was defined as the center of residues that form close contacts with allosteric ligands as described by (Petrassi et al., 2017), i.e., residues 190, 280, 283–286, 289, 293, 311–312, 315–316, 321, 330, 354, 376, 378–379, 382, and 386. Omeprazole is a racemate of two stereoisomers which have been found to have a pKa-value of 7.1, assigned to the dissociation of the protonated pyridine nitrogen (Wu and Delamere, 1997). All four forms of omeprazole were docked independently to MAO-A and TPH1 (to both the active and the postulated allosteric site). To validate our docking protocol, we redocked harmine to MAO-A, obtaining an RMSD of 1.4 Å (Supplementary Figure S1).

### Animal Studies and Drug Administration

Male CD-1 mice (Janvier Labs, Le Genest-St-Isle, France), 3 months of age were used. The animals in the different treatment groups were matched for age. The University of Barcelona’s Committee on Animal Use and Care approved the protocol. Animals were housed and tested in compliance with the guidelines described in the Guide for the Care and Use of Laboratory Animals (Clark et al., 1997) and following the European Union directives (2010/63/EU), FELASA and ARRIVE guidelines. All efforts were made to minimize animal suffering and the number of animals used. All animals were housed in groups of five in standard cages with ad libitum access to food and water and maintained under a 12-h dark/light cycle (starting at 7:30 AM), 22°C temperature, and 66% humidity. Omeprazole was dissolved in 10% DMSO, 50% polyethylene glycol (PEG)-400, and 40% physiological saline. Animals were randomized to receive omeprazole (10 mg/ml) or vehicle, administered i. p. in a volume of 10 µL/g of animal bodyweight, resulting in a dose of 100 mg/kg. Animals received vehicle (n = 22) or omeprazole (n = 24) once daily for four consecutive days. While all treated animals were tested in the tail suspension test, brain tissue from eight of the vehicle-treated and nine of the omeprazole-treated mice was used for subsequent analysis of 5-HT content. Serum samples for 5-HT analysis were drawn from another nine vehicle-treated and eight omeprazole-treated mice.

#### Tail Suspension Test

Immobility time in the tail suspension test is known to be sensitive to manipulations that alter brain 5-HT content (Garcia-Miralles et al., 2016; Palucha-Poniewiera et al., 2017). Animals were subjected to the tail suspension test 20 h after the last administration of vehicle or omeprazole. By use of surgical tape, a cotton thread was affixed to the mouse tail, 1.5 cm from the tip. The cotton thread was fastened to a horizontal metal bar, leaving the mouse suspended in the air, 20 cm above the floor of a cage containing sawdust. Each animal was suspended for 6 min and the time spent immobile during suspension was counted using a stopwatch. Immobility time was defined as the absence of movements of limbs or trunk. Thus, movements of the head alone, such as sniffing, or swinging, pendulum-like motion of the animal resulting from previous bouts of activity, were not considered as mobility. The Mann-Whitney test was used for statistical comparison of immobility time between vehicle- and omeprazole-treated animals.

#### Sample Preparation and High Performance Liquid Chromatography-MS/MS Determination of 5-HT Levels in Brain and Serum

##### Serum Harvesting

Mice were rapidly sacrificed by cervical dislocation and whole blood was obtained by cardiac puncture, collected in clean polypropylene tubes and placed on ice. Serum was prepared by centrifuging the whole blood samples at 15,600 relative centrifugal force (rcf) and 4°C for 20 min. Subsequently, 10 µL serum were mixed with 37.5 µL of TCA (trichloroacetic acid) 30%, 82.5 µL of H_2_O, and 10 µL of 5-HT-*d*_4_ (100 ng/ml sc-473411, Santa Cruz Biotechnology, Dallas, TX). Samples were vortexed for 1 min and incubated overnight at −80°C. Finally, samples were centrifuged at 15,600 rcf and 4°C for 20 min and the supernatants were stored at −80°C until analysis.

##### Brain Homogenates

Mice were rapidly sacrificed by cervical dislocation and one brain hemisphere from each animal dissected in 1 ml of ice-cold H_2_O and homogenized in a 1 ml Potter-Elvehjem glass tube using a homogenizer-stirrer (HS-30E; Witeg Labortechnik GmbH, Wertheim, Germany) with 10 strokes at 700–900 rotations per min. 10 µL of the homogenate was mixed with 10 µL of acetonitrile (Sigma-Aldrich) containing 2% CH_3_COOH (Sigma-Aldrich), 150 µL of H_2_O and 10 µL of 5-HT-*d*_4_ (100 ng/ml sc-473411, Santa Cruz Biotechnology). Samples were vortexed for 1 min and incubated overnight at −80°C, after which samples were centrifuged at 15,600 rcf and 4°C for 20 min. The supernatants were stored at −80°C until analysis.

##### Quantitative Analysis of 5-HT

Quantitative analysis of 5-HT was carried out by liquid chromatography coupled with tandem mass spectrometry (LC-MS/MS) using an Agilent 1,290 Infinity UHPLC chromatograph (Santa Clara, CA) coupled to a 6500 QTRAP mass spectrometer (ABSciex, Framingham, MA) equipped with an Ion Drive Turbo V ion source operating in positive ion mode. The column used was an Acquity HSS T3 1.8 µm (50 × 2.1 mm) at 40°C; autosampler temperature, 4°C; injection volume, 3 μL; flow rate, 0.6 ml m in^−1^. Mobile phase was A) Ultrapure water with 0.1% HCOOH and B) Acetonitrile with 0.1% HCOOH. The gradient program was as follows (t (min), %B) (0, 2), (0.5, 2), (2, 10), (6, 50), (6.2, 95), (7, 95), (7.1, 21), (10, 2). Mass spectrometry detection was performed by using the multiple reaction monitoring (MRM) mode using the following parameters: ion spray voltage, +5500 V; source temperature, 600°C; curtain gas, 20 psi; ion source gas 1 and gas 2, 50 and 50, respectively; collision-activated dissociation gas, High; entrance potential (+/−)10 V. The MRM transitions for 5-HT were 177/160 (Declustering potential DP 20V and collision energy CE15V) for quantitative purposes and 177/115 (DP 20V, CE 37V) for confirmation purposes. 5-HT-*d4* was used as internal standard with a transition of 181/164 (DP 20V, CE 15V). Sample-to-sample differences in recovery, liquid handling, and ionization efficiency were compensated for by normalizing to the amount of 5-HT-*d*4 detected in the samples. Analyst 1.6.2 Software was used for data acquisition and Multi Quant 3.0.1 for data processing; both from ABSciex (Framingham, MA).

A calibration curve was constructed with 5-HT standard solutions between 0.3 and 73.5 nM (brain) or 156–7,364 nM (serum) diluted in acetonitrile with 2% CH_3_COOH. Linear regression was adjusted (1/x or 1/x^2^) in order to have accuracies between 80 and 120% for all the 5-HT standards. 5-HT concentrations were normalized to the mean concentration in samples from vehicle-treated animals. Student’s t-test was used for statistical comparison of 5-HT content between vehicle- and omeprazole-treated animals.

### Data Analysis

The influence of a test compound over the thermal stability of TPH1 was determined as the change in melting temperature, T_m_ $\left(\Delta T_{m}=T_{m}-T_{m/\mathrm{ref}}\right)$, in the presence of 200 µM of the compound. T_m/ref_ is defined as the melting temperature in the presence of vehicle (2% DMSO). GraphPad Prism (version 8; La Jolla, CA, USA) was used for analyzing enzyme inhibition data. The following Eq. 1 was fitted to the data using nonlinear regression, yielding an estimate of the IC_50_:

$$Y=\mathrm{bottom+}\left(\mathrm{Top}-\mathrm{Bottom}\right)/\left(1+10^{X-\mathrm{logIC50}}\right)$$ (1)

where Y is the response as a fraction of 1, X is the logarithm of ligand concentration, Top is the maximum response and Bottom is the minimum response in the presence of ligand.

Relative inhibition of MAO-A was calculated as a percentage using the following Eq. 2:

%Relative inhibition=((Slope of EC−Slope of S)/Slope of EC)×100 (2)

The slopes for all samples, either containing Enzyme Control (EC) or test substance (S), were calculated by dividing the net change in relative fluorescence units (ΔRFU; RFU2–RFU1) by the time interval (Δt; t2 –t1).

*In vitro* assay data are presented as means ± SEM in the Figures and as means and their 95% confidence intervals in the Tables.

## Results

### Identification of Omeprazole as a Tryptophan Hydroxylase 1 Binder

**FIGURE 1:**
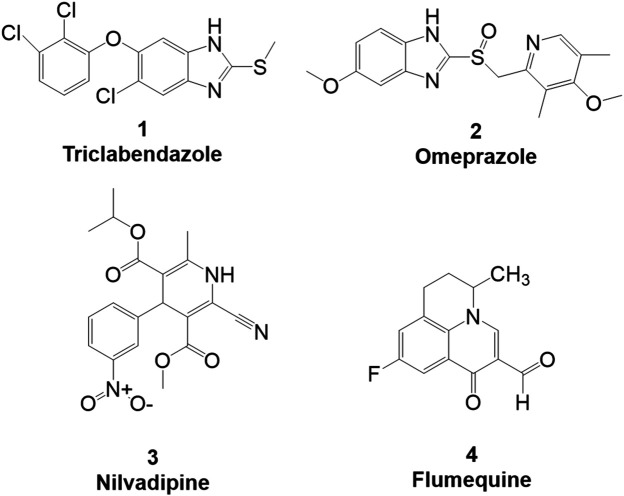
Primary hits identified using DSF. Molecular structure of primary hits identified through HTS using DSF detection and followed by enzymatic activity assay at a final concentration of 100 µM of each tested compound.

As TPH1 is responsible for the majority of 5-HT production in peripheral tissues, we were primarily interested in finding new TPH1 inhibitors, rather than TPH2 inhibitors, due to the potential usefulness of the former in treating dysregulation of peripheral 5-HT. We identified omeprazole, a proton pump inhibitor, as a TPH1 binder through HTS of the Prestwick library using DSF detection as described recently (Betari et al., 2020). The shift in midpoint denaturation temperature, ΔT_m_, was measured in the presence of the various compounds in the Prestwick library in order to find drugs altering the thermal stability of TPH1. The T_m_ of TPH1 was measured by monitoring the fluorescence intensity of a dye, SYPRO orange, the fluorescence of which increases upon interaction with hydrophobic parts of the denatured protein. Hits were identified by determining ΔT_m_ $\left(\Delta T_{m}=T_{m}-T_{m/\mathrm{ref}}\right)$. Under control conditions in the presence of 2% DMSO, the denaturation temperature (T_m/ref_) was 51.8 ± 0.47°C, as recently described (Betari et al., 2020). Compounds which induced a positive ΔT_m_ were considered TPH1 stabilizers, whereas compounds which produced a negative ΔT_m_ were taken to be destabilizers. Among the preliminary hits of the Prestwick library, 37 compounds which stabilized TPH1 with a ΔT_m_ ≥ 3°C or destabilized TPH1 with ΔT_m_ ≤ −3°C were chosen for validation and further investigation in concentration-response DSF experiments. Finally, we employed a TPH activity assay using HPLC with fluorimetric detection to quantify the product, 5-OH-Trp. The effects of 37 preliminary hit compounds on the activity of TPH1 were tested at a concentration of 100 µM. Only four compounds (shown in Figure 1); triclabendazole, omeprazole, nilvadipine, and flumequine, reduced TPH1 activity by more than 50% and were subjected to IC_50_ determination. Omeprazole reduced the activity of TPH1 with an IC_50_ of 3.09 (95% confidence interval; 2.53–3.77) µM, whereas triclabendazole, nilvadipine, and flumequine showed inhibition with very low potency (IC_50_ ∼100 µM).

### Effect of Omeprazole Analogues on Tryptophan Hydroxylase 1 and Tryptophan Hydroxylase 2 Enzymatic Activity

**FIGURE 2:**
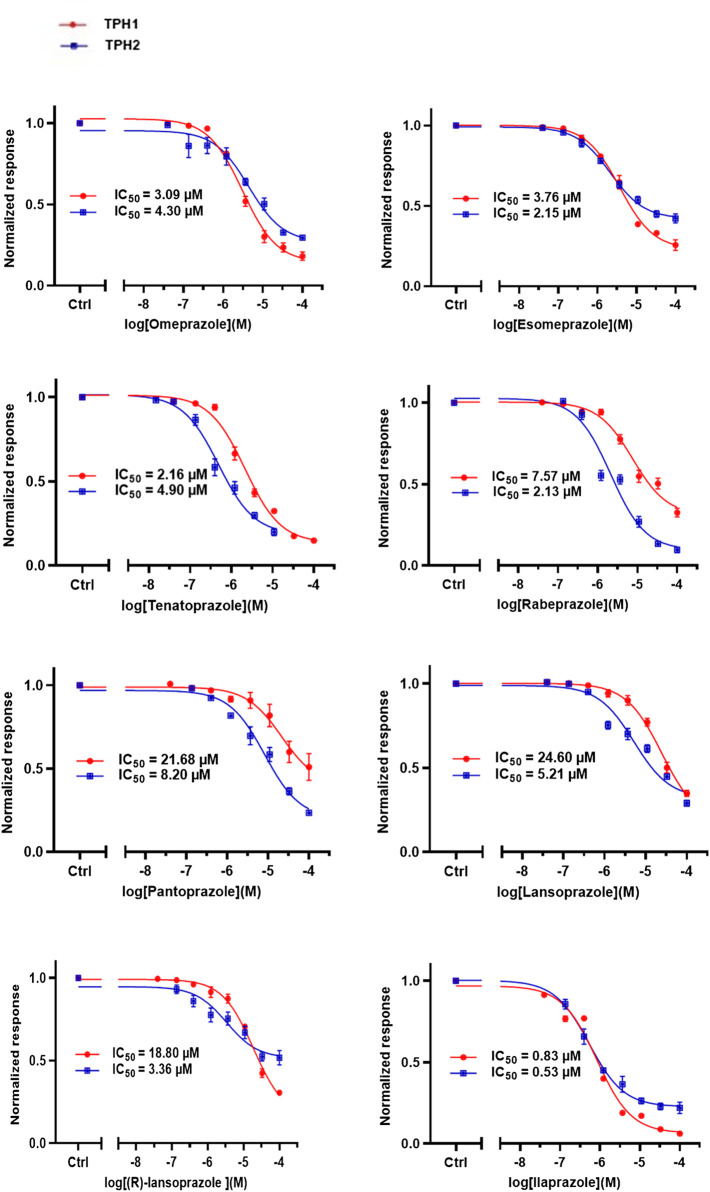
Inhibitory activities of omeprazole and its analogues on TPH1 and TPH2. *In vitro* enzyme activity assays were used to evaluate the effect of proton pump inhibitors to inhibit tryptophan hydroxylase 1 (TPH1); tryptophan hydroxylase 2 (TPH2). Data represent means ± SEM of three separate experiments performed in duplicate.

Following up on the discovery of omeprazole as a TPH1 inhibitor, enzymatic assays of both TPH1 and TPH2 activity were used to investigate the effect of several proton pump inhibitors; esomeprazole, ilaprazole, lansoprazole, R-lanzoprazole, pantoprazole, rabeprazole, and tenatoprazole, which are structural analogues of omeprazole. pCPA and LP533401 were used as reference inhibitors of these two enzymes (Betari et al., 2020). Omeprazole showed inhibitory potency at TPH2 IC_50_ = 4.30 (95% confidence interval; 2.78–6.87) µM similar to that at TPH1 (Table 1). Ilaprazole was revealed to be the most potent TPH inhibitor, with an IC_50_ of 0.83 (95% confidence interval; 0.64–1.8) µM at TPH1 and an IC_50_ of 0.535 (95% confidence interval; 0.39–0.73) µM at TPH2, while esomeprazole, tenatoprazole, and rabeprazole inhibited TPH1 and TPH2 with potencies similar to omeprazole. Pantoprazole, lansoprazole, and (R)-lansoprazole were 6-fold less potent inhibitors of TPH1 than omeprazole, but their potencies were in the same range as omeprazole at TPH2. The effects of these proton pump inhibitors on TPH1 and TPH2 are summarized in Table 1; Figure 2.

**TABLE 1:** Inhibitory activities of omeprazole and its analogues at TPH1 and TPH2, and MAO-A. 1% DMSO was included as a vehicle control.

Name	Structure	TPH1 IC_50_/µM	TPH2 IC_50_/µM	MAO-A IC_50_/µM
pCPA	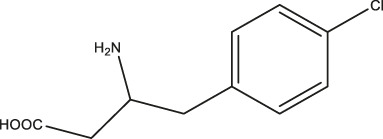	11.25 (7.11–17.84)	5.34 (3.23–8.19)	—
LP533401	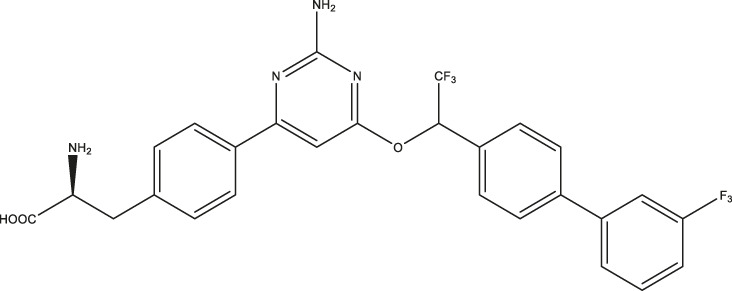	0.41 (0.31–0.53)	0.08 (0.05–0.13)	—
Omeprazole	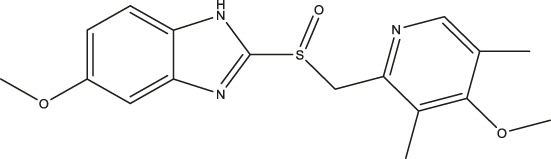	3.09 (2.53–3.77)	4.30 (2.78–6.87)	89.42 (57.48–141.4)
Esomeprazole	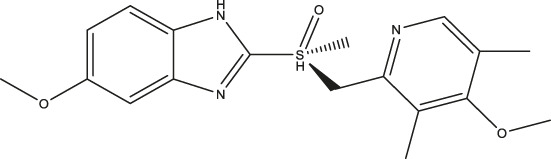	3.76 (3.02–4.67)	2.15 (1.62–2.85)	17.97 (9.15–34.65)
Tenatoprazole	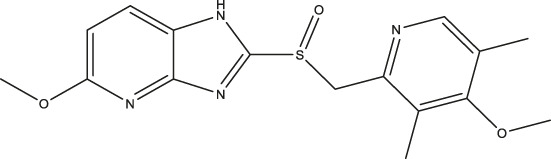	2.16 (1.80–2.60)	4.90 (3.61–6.73)	29.18 (18.02–48.17)
Rabeprazole	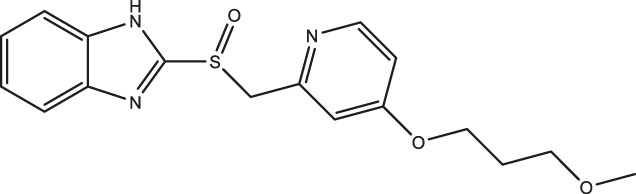	7.57 (5.47–10.56)	2.13 (1.52–3.01)	12.80 (8.02–20.6)
Pantoprazole	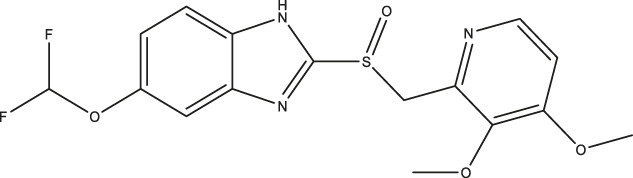	21.68 (9.67–51.83)	8.20 (5.42–12.56)	461.9 (271.0–908.5)
Lansoprazole	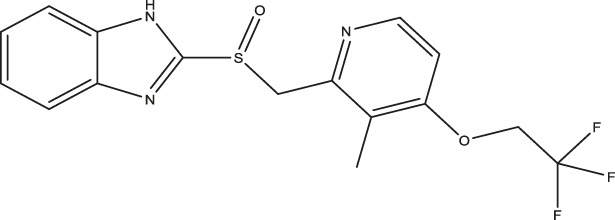	24.60 (18.43–33.25)	5.21 (3.19–8.56)	250.7 (159.4–415.9)
(R)-lansoprazole	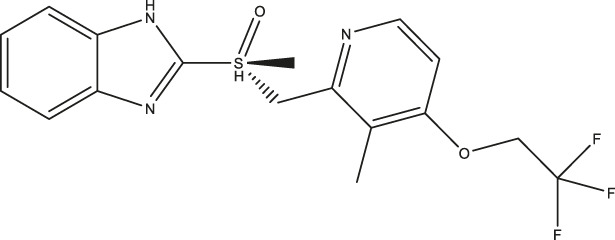	18.80 (14.81–24.04)	3.36 (1.18–8.96)	137.3 (90.83–211.8)
Ilaprazole	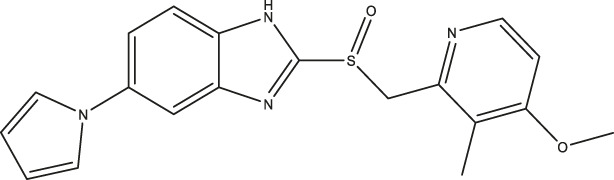	0.83 (0.64–1.8)	0.53 (0.39–0.73)	28.96 (17.16–49.08)

IC_50_ inhibitory concentration (μM) are shown for omeprazole and its analogues. pCPA and LP 533401 were used as reference compounds for TPH1 and TPH2 inhibition. Data represent means of three independent experiments, each performed in duplicate. Values in brackets represent 95% confidence intervals.

### Kinetic Characterization of Tryptophan Hydroxylase 1 Inhibition by Omeprazole

**FIGURE 3:**
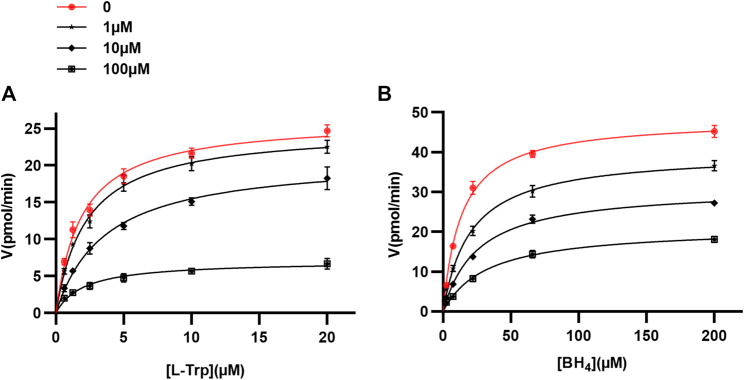
Mechanism of TPH1 inhibition by omeprazole. TPH1 inhibition by omeprazole in the presence of varying concentrations of the substrate; L-Trp **(A)**, or cofactor; BH_4_ **(B)**. The Michaelis-Menten equation was fitted to data using nonlinear regression. The fits are consistent with a decrease in V_max_ with increasing concentrations of compound, while K_M_ remained virtually unchanged.

We chose to focus our further investigation on omeprazole, since it is the most widely used of the proton pump inhibitors. Human doubly truncated TPH1 (ΔNH102-ΔCOOH402) was used for kinetic protein-ligand interaction studies. The enzyme assays were performed as described above. The mechanism of action of omeprazole at TPH1 was investigated by measuring TPH1 inhibition at different concentrations of omeprazole, L-Trp substrate, and tetrahydrobiopterin (BH_4_) cofactor. Kinetic parameters for both L-Trp and BH_4_ were calculated by fitting the Michaelis-Menten equation to the data using nonlinear regression, in the absence and presence of different concentrations of omeprazole (1, 10, and 100 µM). When enzyme activity was measured as a function of substrate (L-Trp) concentration (0.625–20 µM), the concentration of the cofactor (BH_4_) was fixed at 200 µM. As shown in Table 2 and Figure 3A, there was a progressive decrease in V_max_ whereas K_M_ remained essentially unchanged with increasing concentrations of omeprazole. Likewise, when enzyme activity was measured as a function of co-factor concentration (2.4–200 µM) in the presence of a fixed concentration of L-Trp (20 µM), a decreasing V_max_ and a similar K_M_ was again observed as the omeprazole concentration was increased (Table 2; Figure 3B). These results suggest that omeprazole is a non-competitive inhibitor, both with respect to L-Trp and to BH_4_.

**TABLE 2:** Enzyme kinetic parameters of tryptophan hydroxylase one in the absence and presence of omeprazole.

	[Omeprazole] (µM)	V_max_ (µmol/min/mg)	K_M_ (µM)
(A) L-Trp (0.625–20) µM BH_4_ 200 µM	0	26.23 (24.35–28.12)	1.90 (1.43–2.38)
	1	24.96 (23.18–26.74)	2.24 (1.71–2.77)
	10	21.08 (18.84–23.32)	3.61 (2.51–4.72)
	100	7.03 (6.07–7.98)	2.05 (1.10–2.99)
(B) BH_4_ (2.4–200) μM L-Trp 20 µM	0	48.36 (45.94–50.78)	13.66 (11.09–16.24)
	1	39.93 (37.18–42.69)	20.40 (15.57–25.25)
	10	31.06 (28.90–33.21)	25.55 (19.77–31.33)
	100	21.11 (18.96–23.26)	32.43 (22.3–42.55)

(A) TPH1 activity was measured as a function of L-Trp concentration in the absence (DMSO vehicle) or presence of omeprazole (at 1, 10, and 100 µM) and 200 µM BH_4_. (B) TPH1 activity was measured as a function of BH_4_ concentration in the absence (DMSO vehicle) or presence of omeprazole (at 1, 10, and 100 µM) and 20 μM L-Trp. Values in brackets represent 95% confidence intervals for the fit of the Michaelis–Menten equation to data.

### Omeprazole Characterization-Effects on the Other Aromatic Amino Acid Hydroxylases

**FIGURE 4:**
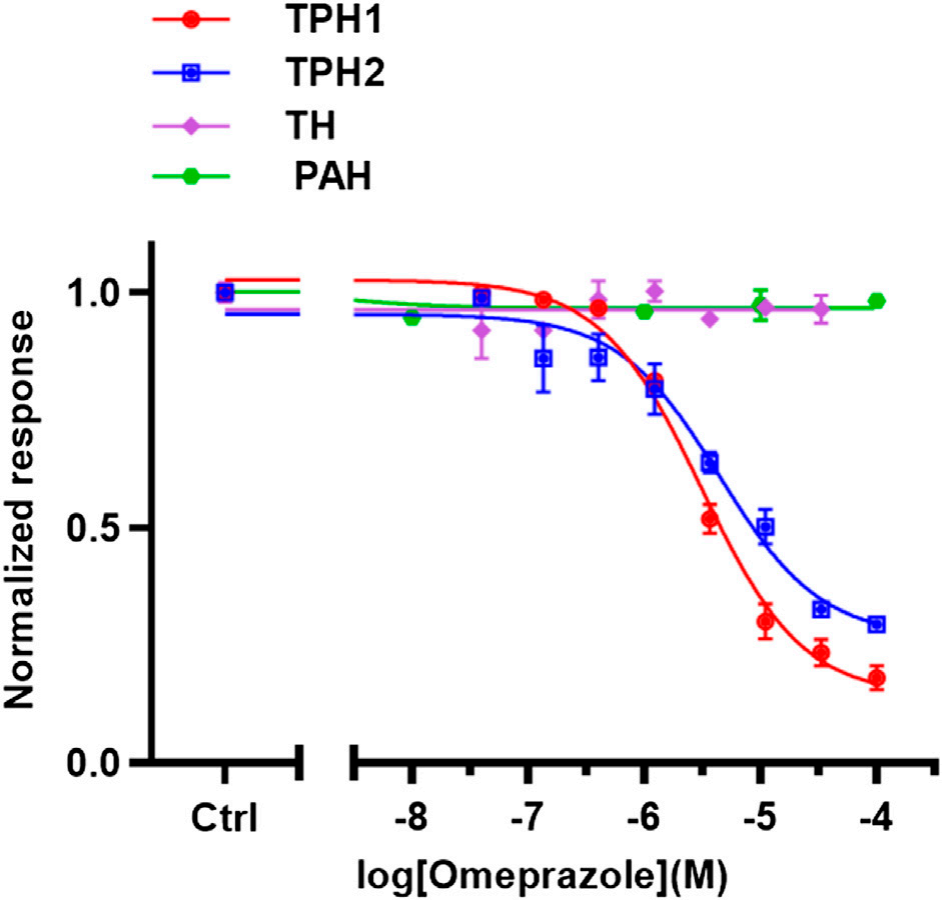
Selectivity of omeprazole to inhibit TPH1 over other aromatic amino acid hydroxylases. *In vitro* enzyme assays were used to evaluate the selectivity of omeprazole to inhibit TPH1 over the other aromatic amino acid hydroxylases; TPH2, TH, and PAH. TPH1, doubly truncated tryptophan hydroxylase 1 (ΔNH102-ΔCOOH402); TPH2, tryptophan hydroxylase two; PAH, phenylalanine hydroxylase, TH tyrosine hydroxylase. The data are expressed as means ± SEM; from three independent experiments, each performed in duplicate.

The potency of omeprazole as a TPH1 inhibitor was compared to those of the benchmark TPH1 inhibitors, pCPA and LP 533401 (data shown in Table 1). The selectivity of omeprazole to inhibit TPH1 over the other aromatic amino acid hydroxylases was also investigated. As described above, omeprazole inhibited TPH2 with similar potency as TPH1. However, no inhibition of PAH or TH was observed up to a concentration of 100 µM (Figure 4). Thus, omeprazole inhibition of TPH1 and TPH2 was highly selective over the two other aromatic amino acid hydroxylases, PAH and TH. Finally, the inhibitory potency of omeprazole at full-length TPH1 was studied and found to be virtually identical to that observed in experiments with doubly truncated TPH1 (Supplementary Figure S2).

### Effect of Omeprazole and Its Analogues on Monoamine Oxidase-A Enzymatic Activity

**FIGURE 5:**
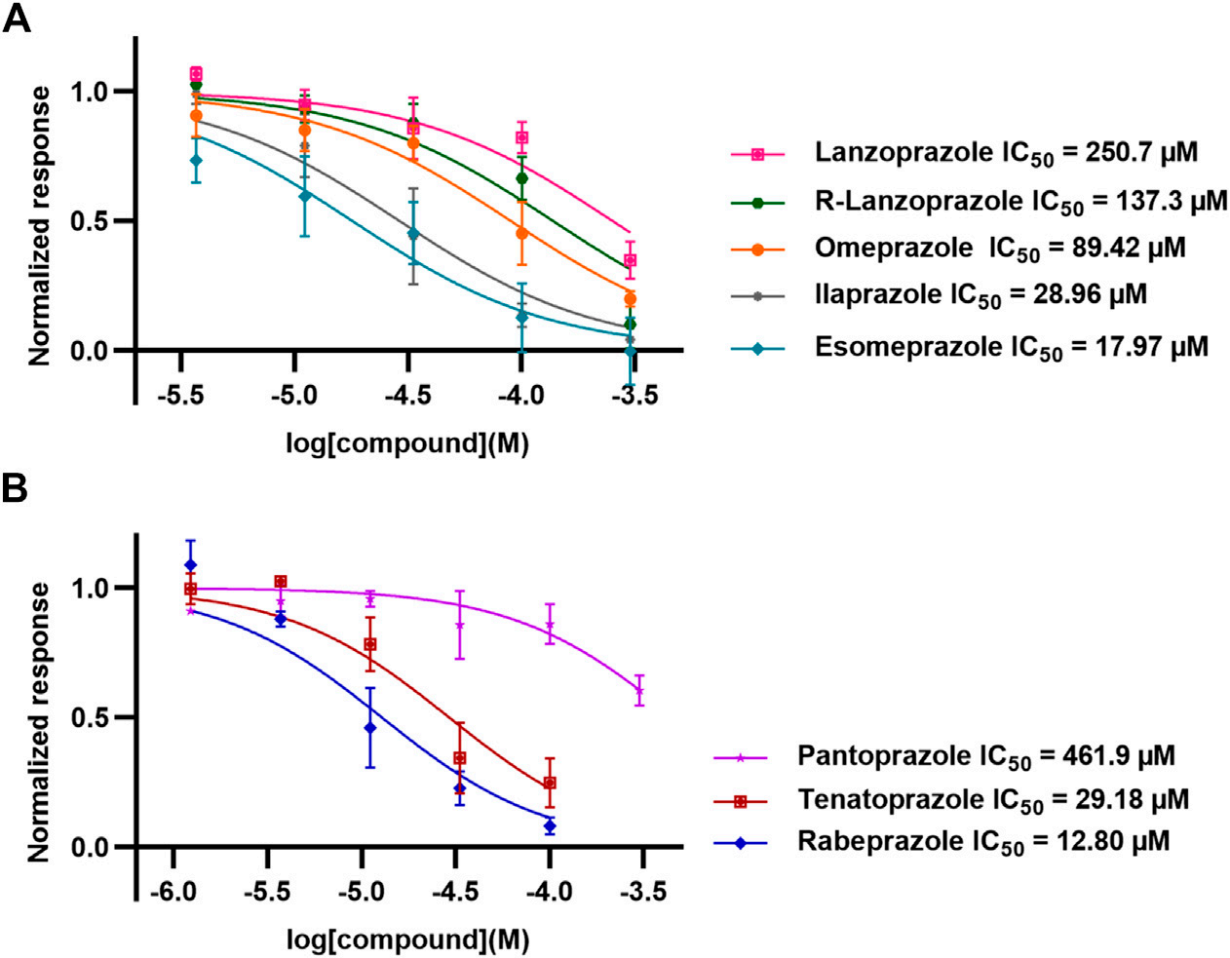
Inhibitory activities of omeprazole and its analogues at MAO-A. *In vitro* enzyme activity assays were used to evaluate the potencies of omeprazole and its analogues to inhibit monoamine oxidase-A (MAO-A). Data represent means ± SEM of three separate experiments.

The effects of omeprazole, esomeprazole, ilaprazole, lansoprazole, R-lanzoprazole, pantoprazole, rabeprazole, and tenatoprazole on MAO-A enzymatic activity were tested using a fluorescence-based MAO-A activity assay measuring the formation of H_2_O_2_ as a byproduct of the enzymatic activity (Figure 5).

### Docking Studies of Omeprazole Binding to Tryptophan Hydroxylase1 and Monoamine Oxidase-A

**FIGURE 6:**
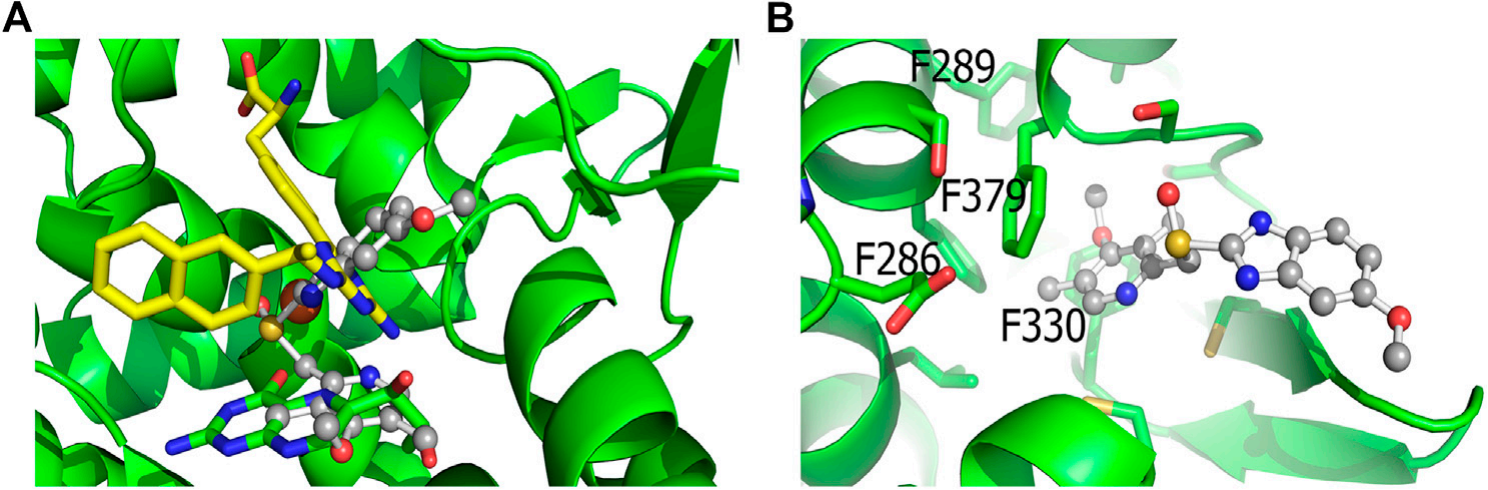
Docking of omeprazole to TPH1. **(A)** Docking of omeprazole to the active site of TPH1. Omeprazole is shown in ball and stick representation with carbons colored gray while the biopterin cofactor is shown in sticks with green carbons. The active site iron is shown as an orange sphere. Superimposed is also the inhibitor from Lexicon Pharmaceuticals (LP-521834) in sticks with yellow carbons. Omeprazole partially overlaps with the binding pockets of both the cofactor and LP-521834 when docked to the active site of TPH1. **(B)** Docking of omeprazole to the postulated allosteric site of TPH1. Omeprazole (shown as ball and sticks) docks close to the surface of the postulated allosteric pocket of TPH1, near the four phenylalanines defining the entrance of the site (F286, F289, F330 and F379), shown as green sticks in the figure.

**FIGURE 7:**
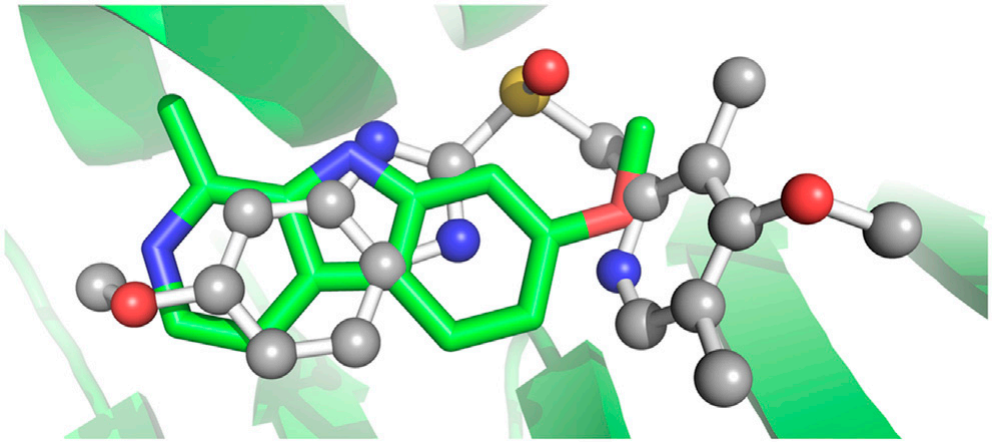
Docking of Omeprazole to MAO-A. The docking pose of omeprazole with the highest docking score is shown with in ball and stick with gray carbons. Also shown is the binding pose of harmine in sticks with green carbons. The docking pose of omeprazole overlaps entirely with harmine in the binding pocket of MAO-A.

The protonated S-stereoisomer of omeprazole was predicted to have the highest binding affinity to both MAO-A and TPH1, considering both the active site of TPH1 (Figure 6A) and its previously described allosteric site (Petrassi et al., 2017). Omeprazole overlaps with the binding site of the known inhibitor, harmine, present in the original crystal structure (Son et al., 2008), when docked to MAO-A (Figure 7). Omeprazole was predicted to have higher affinity for MAO-A than for TPH1, and to have a slight preference for the allosteric site over the active site in TPH1 (Table 3).

**TABLE 3:** Docking score (estimated binding affinities) of omeprazole and harmine to TPH1 and MAO-A.

	TPH1-active site	TPH1-allosteric site	MAO-A
Omeprazole^a^	−8.2	−8.6	−11.1

a S-isomer of protonated omeprazole. Docking score is a crude estimate of the free energy of binding. The more negative docking score, the higher the predicted binding affinity.

### *In vivo* Studies

Omeprazole has commonly been administered i. p. to mice at doses of 20–150 mg/kg (Cowan et al., 2005). We were interested in finding out how the seemingly opposing actions of omeprazole, as a TPH inhibitor on the one hand and an MAO-A inhibitor on the other, might affect its *in vivo* profile. We thus administered omeprazole (100 mg/kg, i. p.) to mice once daily for four days. Twenty hours after the last dose, animals were subjected to the tail suspension test; a behavioral readout which is sensitive to CNS 5-HT levels and commonly used to assess antidepressant-like activity of experimental compounds. Compared to vehicle controls, mice receiving omeprazole spent significantly less time immobile during the 6 min tail suspension trial (*p* = 0.028, Mann-Whitney test; Supplementary Figure S3).

**FIGURE 8:**
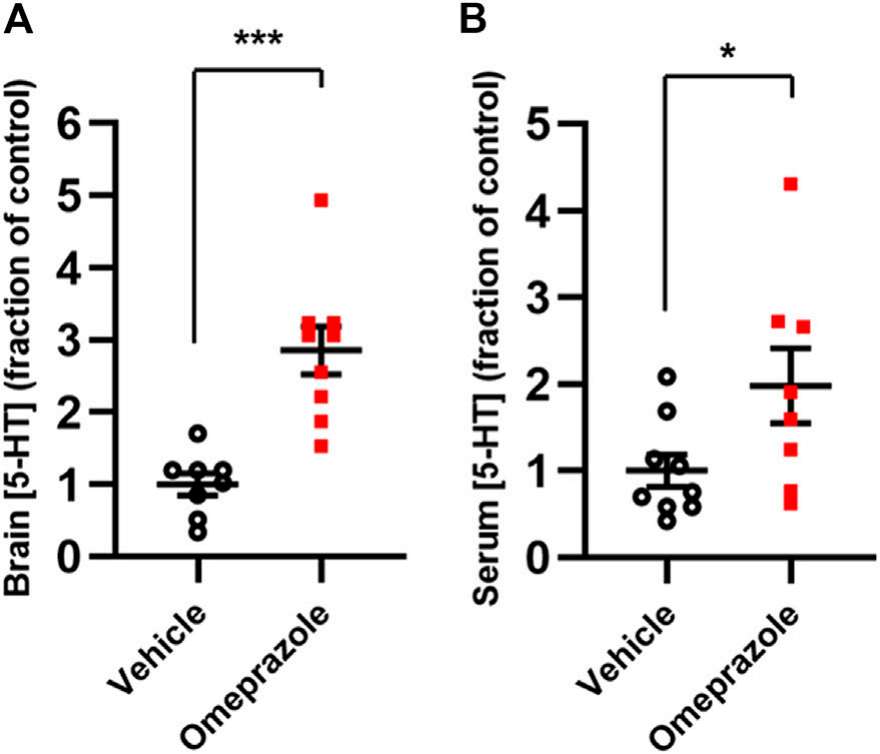
*In vivo* effects of 100 mg/kg omeprazole on 5-HT levels. 5-HT contents, as determined by LC-MS, in whole-brain tissue (A; vehicle, n = 8; omeprazole, n = 9) and serum (B; vehicle, n = 9; omeprazole, n = 8) in mice treated with omeprazole (100 mg/kg, i. p.) or vehicle once daily for 4 days *, *p* < 0.05; ***, *p* < 0.001, Student’s t-test.

For analysis of brain and serum 5-HT content, the animals were sacrificed by cervical dislocation immediately after behavioral testing and brain tissue and serum were harvested. Omeprazole-treated animals displayed significantly higher 5-HT content in both brain (Figure 8A) and serum (Figure 8B) compared to vehicle controls.

## Discussion and Conclusion

In the present investigation, we showed that the proton pump inhibitor, omeprazole, as well as several analogues, inhibit TPH1 and TPH2 with low micromolar potency. Omeprazole was the first proton pump inhibitor when approved for clinical use in 1988 (Lindberg and Carlsson, 2006). It has been designated an essential medicine by the World Health Organization, was the most commonly prescribed drug in the United States in 2017 and can be obtained without a prescription in many countries (WHO, 2019). Although, for example, ilaprazole showed higher potency to inhibit TPH1, we chose to focus our further investigative efforts on omeprazole, given its widespread use.

Kinetic measurements of omeprazole inhibition of TPH1 suggest that omeprazole binds in a noncompetitive fashion with respect to both the cofactor and the substrate. This may suggest that omeprazole does not bind to the tryptophan or cofactor binding sites, but rather to an allosteric site in the protein. Our computational docking results are also compatible with an allosteric mode of action (see below). Allosteric TPH1 inhibitors are of potential interest for further development, as such ligands can be expected to bind outside the conserved active site and thus, potentially, show greater selectivity between the different aromatic amino acid hydroxylases. Selective TPH1 inhibitors are of interest from a medicinal chemistry perspective, since elevated peripheral 5-HT is an important component of several disease conditions, such as carcinoid syndrome; a malignancy-related hyperserotonergic state characterized by severe diarrhea, as well as irritable bowel syndrome and infectious and autoimmune states associated with gut inflammation, including inflammatory bowel disease (Manocha and Khan, 2012; Margolis et al., 2014; Kim et al., 2015). TPH1 inhibitors with potencies in the nanomolar range have been reported by Lexicon Pharmaceuticals, out of which one compound, telotristat, was recently approved for treatment of carcinoid syndrome (Jin et al., 2009; Ouyang et al., 2012). The Lexicon compounds, however, are orthosteric inhibitors structurally related to pCPA and behave, as expected, competitively with regards to the substrate (Cianchetta et al., 2010). We recently reported the benzisothiazolinone, PBIT, and two related compounds, as TPH inhibitors with similarly non-competitive actions at TPH1(Betari et al., 2020) These compounds displayed excellent selectivity toward TH, similar to omeprazole, but showed some inhibition of PAH at high micromolar concentrations.

As already mentioned, the similarity between the active sites of the two TPH isoforms, TH, and PAH means that achieving ligand specificity has been difficult. This fact has therapeutic implications, since off-target inhibition of PAH may potentially lead to neurotoxic accumulation of its substrate, phenylalanine. It is therefore noteworthy that the inhibitory effect of omeprazole was highly specific for TPH1 and TPH2 over both TH and PAH, with virtually no inhibition observed at the latter two enzymes at concentrations up to 100 µM. Elaboration of the proton pump inhibitor scaffold may thus be of relevance for future studies, in order to develop more potent TPH inhibitors devoid of activity toward TH and PAH.

Esomeprazole, the S-enantiomer of omeprazole, was recently shown to display high micromolar potency in inhibiting MAO-A (Petzer et al., 2013). We validated this finding by testing omeprazole and its analogues in an MAO-A inhibition assay. While esomeprazole showed the expected inhibitory potency with an IC_50_ of 17.97 (95% confidence interval; 9.15–34.65) µM (compared to 23 µM in Petzer et al., 2013), racemic omeprazole was somewhat less potent with an IC_50_ of 89.42 (95% confidence interval; 57.48–141.4) µM. Among the other proton pump inhibitors tested, rabeprazole was the most potent with an IC_50_ of 12.80 (95% confidence interval; 8.02–20.6) µM and pantoprazole the least potent with an IC_50_ of 461.9 (95% confidence interval; 271.0–908.5) µM.

The potency of proton pump inhibitors at their therapeutic target, the gastric H^+^/K^+^-ATPase, is reportedly in the low micromolar range and thus similar to the IC_50_ s at TPH1 and TPH2 observed in the present study (Beil et al., 1992). In particular, given that many studies in mice have been conducted with proton pump inhibitors doses ranging from 20 to 150 mg/kg (Cowan et al., 2005; Fontecha-Barriuso et al., 2020), the *in vivo* actions of these compounds at both TPH1/2 and MAO-A should also be considered.

In agreement with our experimental findings, docking studies indicate that the protonated S-enantiomer of omeprazole, esomeprazole, has the highest binding affinity for both TPH1 and MAO-A. Omeprazole overlaps with the binding of the known MAO-A inhibitor, harmine, in MAO-A and is estimated to have similar binding affinities to the active site and the allosteric binding site (Petrassi et al., 2017) of TPH1. When docked to the active site of TPH1, omeprazole partly occupies the cofactor and substrate binding pocket (LP-521834 demonstrates a similar binding mode). The docking results are not decisive in which of the two TPH1 pockets, the active site or the allosteric site, is the most favorable for omeprazole to interact with. However, as omeprazole inhibits TPH1 in a non-competitive manner, it would seem more likely to interact with TPH1 outside of the active site.

Use of proton pump inhibitors has been associated with a higher incidence of clinical depression (Huang et al., 2018; Laudisio et al., 2018), which may be congruent with 5-HT depletion resulting from the TPH2 inhibitory action observed in the present study. On the other hand, MAO-A inhibitors are used clinically for their antidepressant actions, presumably mediated via 5-HT elevation. In order to explore the net *in vivo* effect of these presumably opposing actions on 5-HT metabolism, we administered omeprazole i. p. to mice at a dose of 100 mg/kg for four consecutive days. Somewhat surprisingly, given the lower *in vitro* potency of omeprazole at MAO-A compared to TPH1/2, omeprazole-treated animals had significantly higher 5-HT levels both in serum and in whole-brain tissue relative to vehicle-treated controls. In agreement with a positive effect on brain 5-HT levels, mice treated with omeprazole spent significantly less time immobile in the tail suspension test, compared to control mice. Thus, it appears that, at least at the dose used in the present study, the net outcome of the opposing effects of omeprazole is to increase 5-HT *in vivo.* In this context, it is interesting to note that there has been a recent case report of serotonin syndrome in a patient receiving omeprazole in combination with the SSRI, citalopram (Tsamatsoulis et al., 2018), which would be congruent with the MAO-A inhibition described here and by (Petzer et al., 2013). Thus, omeprazole may have different effects on the 5-HT system depending on dose and co-administration of other serotonergic drugs. As mentioned above, besides its central actions, 5-HT has a prominent role in exacerbating gut inflammation (Manocha and Khan, 2012; Margolis et al., 2014; Kim et al., 2015) and proton pump inhibitor-mediated actions at TPH1 and MAO-A could thus have either beneficial or detrimental consequences, depending on the net effect on local 5-HT concentrations. For example, when administered for the treatment of *Helicobacter pylori*-related ulcers or gastroesophageal reflux disease (Herszenyi et al., 2020), TPH1 inhibition might be expected to reduce inflammation through a reduction of 5-HT in the gastrointestinal mucosa, whereas MAO-A inhibition would produce an opposite, potentially pro-inflammatory effect.

The 5-HT found in blood (both in serum and in platelets) is mainly produced by the enterochromaffin cells in the gut and taken up by platelets via the 5-HT transporter (SERT). It could be hypothesized that SERT blockade, rather than MAO-A inhibition, would be responsible for the elevation of serum 5-HT observed here. While we have not assessed any such putative actions of omeprazole on SERT in the present work, several previous observations argue against this possibility. SERT KO mice show virtually undetectable levels of 5-HT in blood (Chen et al., 2001) and while acute administration of SERT blockers transiently (4 h) elevated plasma 5-HT, subchronic (7 or 14 days) treatment with SERT blockers did not modify plasma 5-HT (Ortiz and Artigas, 1992), suggesting that 5-HT released from the enterochromaffin cells is effectively degraded or removed if not taken up by platelets. Degradation by MAO-A located in the liver and vascular endothelial cells would be a likely mechanism for 5-HT removal (Ortiz and Artigas, 1992). Furthermore, the total tissue content of brain 5-HT (as measured here) has been found to be decreased in SERT KO rats (Homberg et al., 2007).

In contrast, subchronic administration of the MAO-A inhibitor, clorgyline, increased plasma 5-HT levels (Garcia-Miralles et al., 2016). Thus, while caution is always warranted when extrapolating *in vitro* data to the *in vivo* situation, we would tend to speculate that the increase in blood and brain 5-HT content observed here following subchronic treatment with omeprazole is most likely a consequence of MAO-A inhibition. Finally, while we have not studied the involvement of 5-HT receptors in the actions of omeprazole, 5-HT_2B_ receptors are also known to play a role in the regulation of plasma 5-HT levels (Callebert et al., 2006).

The tail suspension test was used here because it is known to be sensitive to manipulations which alter brain 5-HT content (Garcia-Miralles et al., 2016; Palucha-Poniewiera et al., 2017). However, in the context of translatability it should be pointed out that use of this single behavioral test is a limitation of the present study and that the use of additional paradigms (such as the forced swim test, or genetic- or chronic stress models of depression) would be necessary in order to firmly establish an antidepressant-like effect of omeprazole (Wang et al., 2017)**.** Furthermore, such antidepressant-like effects are not only influenced by brain 5-HT content, but is a consequence of extracellular (rather than whole brain) 5-HT and the activation states of multiple subtypes of 5-HT receptors (Pytka et al., 2016b) as well as of other neurotransmitter receptors (Pytka et al., 2016a). Thus, the fact that we have not studied any potential effect of omeprazole on 5-HT receptors is a further limitation of the present work.

In summary, omeprazole and other structurally related proton pump inhibitors reduced the catalytic activity of TPH1 and TPH2 in the low micromolar range, whereas MAO-A inhibition was observed at higher micromolar concentrations. Interestingly, omeprazole did not appreciably inhibit TH nor PAH at concentrations as high as 100 µM. When administered at 100 mg/kg, omeprazole increased 5-HT concentrations in serum and brain tissue and decreased immobility time in the tail suspension test. Serotonergic actions should thus be considered when evaluating the *in vivo* effects of proton pump inhibitors. Interestingly, some of these compounds appeared to have quite different relative potencies against TPH1, TPH2, and MAO-A (Table 1; Figure 2; Figure 5). This indicates that it may be possible to develop more selective inhibitors targeting either one of the TPH isoforms, using the proton pump inhibitor scaffold as a point of departure. Future investigations of the structure-activity relationships of proton pump inhibitor-like scaffolds at TPH1, TPH2, and MAO-A may result in new TPH inhibitors with increased selectivity over TH and PAH.

## Data Availability Statement

The raw data supporting the conclusions of this article will be made available by the authors, without undue reservation.

## Ethics Statement

The animal study was reviewed and approved by The University of Barcelona’s Committee on Animal Use and Care.

## Author Contributions

NB designed and performed *in vitro* enzyme experiments, analyzed data, and wrote the first draft of the manuscript. KS designed and performed *in vivo* experiments and contributed to data analysis and to writing the first draft. XM performed LC-MS experiments and wrote the corresponding methods section. HG-M performed LC-MS experiments. OJ supervised LC-MS experiments and performed related data analysis. KT performed and analyzed molecular docking studies and contributed to manuscript writing. FC supervised the *in vivo* work, supplied laboratory resources and funding, and contributed to manuscript writing. JH supervised the project, supplied laboratory resources and funding, and contributed to manuscript writing. All authors approved the final version of the manuscript.

## Funding

This study was financed by grants from the European Union’s Horizon 2020 research and innovation program under Grant Agreement No. 667302 (CoCA), the Research Council of Norway (Grant 249951) and Stiftelsen K. G. Jebsen (SKGJ-MED02) to JH. FC was funded by Ministerio de Ciencia, Innovación y Universidades–Agencia Estatal de Investigación/FEDER (SAF 2017–87349-R). KS was funded by the Wallenberg Center for Molecular Medicine at Umeå University.

## Conflict of Interest

JH has served as a speaker for Eli-Lilly, HB Pharma, Biocodex, Takeda, Medice, and Shire.

The remaining authors declare that the research was conducted in the absence of any commercial or financial relationships that could be construed as a potential conflict of interest.

## Abbreviations

**Abbreviations:** 5-HT, serotonin; TPH, tryptophan hydroxylase; MAO-A, monoamine oxidase-A; TH, tyrosine hydroxylase; PAH, phenylalanine hydroxylase; BH_4_, (6R)-L-erythro-5,6,7,8-tetrahydrobiopterin; DSF, differential scanning fluorimetry; HTS, high-throughput screening; L-Trp, _l_-tryptophan; 5-OH-Trp, 5-hydroxy-tryptophan; HPLC, high performance liquid chromatography; LC-MS, liquid chromatography-mass spectrometry; IC_50_, inhibitory concentration 50%; SEM, standard error of the mean

## References

[B1] Aubi O. Flydal M. I. Zheng H. Skjaerven L. Rekand I. Leiros H. K. (2015). Discovery of a specific inhibitor of pyomelanin synthesis in *Legionella pneumophila* . J. Med. Chem. 58, 8402–8412. 10.1021/acs.jmedchem.5b01589 26458252

[B2] Beil W. Staar U. Sewing K. F. (1992). Pantoprazole: a novel H+/K(+)-ATPase inhibitor with an improved pH stability. Eur. J. Pharmacol. 218, 265–271. 10.1016/0014-2999(92)90178-7 1330598

[B3] Berger M. Gray J. A. Roth B. L. (2009). The expanded biology of serotonin. Annu. Rev. Med. 60, 355–366. 10.1146/annurev.med.60.042307.110802 19630576 PMC5864293

[B4] Betari N. Sahlholm K. Ishizuka Y. Teigen K. Haavik J. (2020). Discovery and biological characterization of a novel scaffold for potent inhibitors of peripheral serotonin synthesis. Future Med. Chem. 12, 1461–1474. 10.4155/fmc-2020-0127 32752885

[B5] Bezem M. T. Baumann A. Skjaerven L. Meyer R. Kursula P. Martinez A. (2016). Stable preparations of tyrosine hydroxylase provide the solution structure of the full-length enzyme. Sci. Rep. 6, 30390 10.1038/srep30390 27462005 PMC4961952

[B6] Callebert J. Esteve J. M. Herve P. Peoc'h K. Tournois C. Drouet L. (2006). Evidence for a control of plasma serotonin levels by 5-hydroxytryptamine(2B) receptors in mice. J. Pharmacol. Exp. Therapeut. 317, 724–731. 10.1124/jpet.105.098269 16461587

[B7] Chen J. J. Li Z. Pan H. Murphy D. L. Tamir H. Koepsell H. (2001). Maintenance of serotonin in the intestinal mucosa and ganglia of mice that lack the high-affinity serotonin transporter: abnormal intestinal motility and the expression of cation transporters. J. Neurosci. 21, 6348–6361. 10.1523/JNEUROSCI.21-16-06348.2001 11487658 PMC6763151

[B8] Cianchetta G. Stouch T. Yu W. Shi Z. C. Tari L. W. Swanson R. V. (2010). Mechanism of inhibition of novel tryptophan hydroxylase inhibitors revealed by co-crystal structures and kinetic analysis. Curr. Chem. Genom. 4, 19–26. 10.2174/1875397301004010019 10.2174/1875397301004010019 PMC2885594 20556201

[B9] Clark J. D. Gebhart G. F. Gonder J. C. Keeling M. E. Kohn D. F. (1997). Special report: the 1996 guide for the care and use of laboratory animals. ILAR J. 38, 41–48. 10.1093/ilar.38.1.41 11528046

[B10] Corsello S. M. Bittker J. A. Liu Z. Gould J. Mccarren P. Hirschman J. E. (2017). The drug repurposing hub: a next-generation drug library and information resource. Nat. Med. 23, 405–408. 10.1038/nm.4306 28388612 PMC5568558

[B11] Cote F. Fligny C. Bayard E. Launay J. M. Gershon M. D. Mallet J. (2007). Maternal serotonin is crucial for murine embryonic development. Proc. Natl. Acad. Sci. U. S. A. 104, 329–334. 10.1073/pnas.0606722104 17182745 PMC1713169

[B12] Cowan A. Earnest D. L. Ligozio G. Rojavin M. A. (2005). Omeprazole-induced slowing of gastrointestinal transit in mice can be countered with tegaserod. Eur. J. Pharmacol. 517, 127–131. 10.1016/j.ejphar.2005.05.041 15972210

[B13] De Colibus L. Li M. Binda C. Lustig A. Edmondson D. E. Mattevi A. (2005). Three-dimensional structure of human monoamine oxidase A (MAO A): relation to the structures of rat MAO A and human MAO B. Proc. Natl. Acad. Sci. U. S. A. 102, 12684–12689. 10.1073/pnas.0505975102 16129825 PMC1200291

[B14] Engelman K. Lovenberg W. Sjoerdsma A. (1967). Inhibition of serotonin synthesis by para-chlorophenylalanine in patients with the carcinoid syndrome. N. Engl. J. Med. 277, 1103–1108. 10.1056/NEJM196711232772101 6054996

[B15] Finberg J. P. Rabey J. M. (2016). Inhibitors of MAO-A and MAO-B in psychiatry and neurology. Front. Pharmacol. 7, 340 10.3389/fphar.2016.00340 27803666 PMC5067815

[B16] Flydal M. I. Chatfield C. H. Zheng H. Gunderson F. F. Aubi O. Cianciotto N. P. (2012). Phenylalanine hydroxylase from *Legionella pneumophila* is a thermostable enzyme with a major functional role in pyomelanin synthesis. PloS One 7, e46209 10.1371/journal.pone.0046209 23049981 PMC3458870

[B17] Fontecha-Barriuso M. Martin-Sanchez D. Martinez-Moreno J. M. Cardenas-Villacres D. Carrasco S. Sanchez-Nino M. D. (2020). Molecular pathways driving omeprazole nephrotoxicity. Redox Biol 32, 101464 10.1016/j.redox.2020.101464 32092686 PMC7038587

[B18] Garcia-Miralles M. Ooi J. Ferrari Bardile C. Tan L. J. George M. Drum C. L. (2016). Treatment with the MAO-A inhibitor clorgyline elevates monoamine neurotransmitter levels and improves affective phenotypes in a mouse model of Huntington disease. Exp. Neurol. 278, 4–10. 10.1016/j.expneurol.2016.01.019 26825854

[B19] Gillman P. K. (2006). A review of serotonin toxicity data: implications for the mechanisms of antidepressant drug action. Biol. Psychiatr. 59, 1046–1051. 10.1016/j.biopsych.2005.11.016 16460699

[B20] Gillman P. K. (2011). Advances pertaining to the pharmacology and interactions of irreversible nonselective monoamine oxidase inhibitors. J. Clin. Psychopharmacol. 31, 66–74. 10.1097/JCP.0b013e31820469ea 21192146 10.1097/JCP.0b013e31820469ea

[B21] Goldberg D. R. De Lombaert S. Aiello R. Bourassa P. Barucci N. Zhang Q. (2017). Optimization of spirocyclic proline tryptophan hydroxylase-1 inhibitors. Bioorg. Med. Chem. Lett 27, 413–419. 10.1016/j.bmcl.2016.12.053 28041831

[B22] Haavik J. Flatmark T. (1980). Rapid and sensitive assay of tyrosine 3-monooxygenase activity by high-performance liquid chromatography using the native fluorescence of DOPA. J. Chromatogr. 198, 511–515. 10.1016/S0021-9673(00)80522-1 6108329

[B23] Herszenyi L. Bakucz T. Barabas L. Tulassay Z. (2020). Pharmacological approach to gastric acid suppression: past, present, and future. Dig. Dis. 38, 104–111. 10.1159/000505204 31846972

[B24] Homberg J. R. Olivier J. D. Smits B. M. Mul J. D. Mudde J. Verheul M. (2007). Characterization of the serotonin transporter knockout rat: a selective change in the functioning of the serotonergic system. Neuroscience 146, 1662–1676. 10.1016/j.neuroscience.2007.03.030 17467186

[B25] Howden C. W. (1991). Clinical pharmacology of omeprazole. Clin. Pharmacokinet. 20, 38–49. 10.2165/00003088-199120010-00003 2029801

[B26] Huang W. S. Bai Y. M. Hsu J. W. Huang K. L. Tsai C. F. Su T. P. (2018). Use of proton pump inhibitors and risk of major depressive disorder: a nationwide population-based study. Psychother. Psychosom. 87, 62–64. 10.1159/000485190 29306949

[B27] Jin H. Cianchetta G. Devasagayaraj A. Gu K. Marinelli B. Samala L. (2009). Substituted 3-[4-(1,3,5-triazin-2-yl)-phenyl]-2-aminopropanoic acids as novel tryptophan hydroxylase inhibitors. Bioorg. Med. Chem. Lett 19, 5229–5232. 10.1016/j.bmcl.2009.07.005 19631532

[B28] Keiser M. J. Setola V. Irwin J. J. Laggner C. Abbas A. I. Hufeisen S. J. (2009). Predicting new molecular targets for known drugs. Nature 462, 175–181. 10.1038/nature08506 19881490 PMC2784146

[B29] Kim J. J. Wang H. Terc J. D. Zambrowicz B. Yang Q. M. Khan W. I. (2015). Blocking peripheral serotonin synthesis by telotristat etiprate (LX1032/LX1606) reduces severity of both chemical- and infection-induced intestinal inflammation. Am. J. Physiol. Gastrointest. Liver Physiol. 309, G455-465. 10.1152/ajpgi.00299.2014 26206858

[B30] Laudisio A. Antonelli Incalzi R. Gemma A. Giovannini S. Lo Monaco M. R. Vetrano D. L. (2018). Use of proton-pump inhibitors is associated with depression: a population-based study. Int. Psychogeriatr. 30, 153–159. 10.1017/S1041610217001715 28899441

[B31] Lindberg P. Carlsson E. (2006). “Esomeprazole in the framework of proton‐pump inhibitor development,” in Analogue-based drug discovery. Editors Fischer J. Ganellin D. C. R. (Wiley‐VCH Verlag GmbH & Co. KGaA), 81–113.

[B32] Lo M. C. Aulabaugh A. Jin G. Cowling R. Bard J. Malamas M. (2004). Evaluation of fluorescence-based thermal shift assays for hit identification in drug discovery. Anal. Biochem. 332, 153–159. 10.1016/j.ab.2004.04.031 15301960

[B33] Manocha M. Khan W. I. (2012). Serotonin and GI disorders: an update on clinical and experimental studies. Clin. Transl. Gastroenterol. 3, e13 10.1038/ctg.2012.8 23238212 10.1038/ctg.2012.8 PMC3365677

[B34] Margolis K. G. Stevanovic K. Li Z. Yang Q. M. Oravecz T. Zambrowicz B. (2014). Pharmacological reduction of mucosal but not neuronal serotonin opposes inflammation in mouse intestine. Gut 63, 928–937. 10.1136/gutjnl-2013-304901 23749550 PMC4034681

[B35] Markham A. (2017). Telotristat ethyl: first global approval. Drugs 77, 793–798. 10.1007/s40265-017-0737-x 28382568

[B36] Matthes S. Bader M. (2018). Peripheral serotonin synthesis as a new drug target. Trends Pharmacol. Sci. 39, 560–572. 10.1016/j.tips.2018.03.004 29628275

[B37] Mckinney J. Knappskog P. M. Haavik J. (2005). Different properties of the central and peripheral forms of human tryptophan hydroxylase. J. Neurochem. 92, 311–320. 10.1111/j.1471-4159.2004.02850.x 15663479

[B38] Mckinney J. Knappskog P. M. Pereira J. Ekern T. Toska K. Kuitert B. B. (2004). Expression and purification of human tryptophan hydroxylase from *Escherichia coli* and Pichia pastoris. Protein Expr. Purif. 33, 185–194. 10.1016/j.pep.2003.09.014 14711505

[B39] Meyer J. H. Ginovart N. Boovariwala A. Sagrati S. Hussey D. Garcia A. (2006). Elevated monoamine oxidase a levels in the brain: an explanation for the monoamine imbalance of major depression. Arch. Gen. Psychiatr. 63, 1209–1216. 10.1001/archpsyc.63.11.1209 17088501

[B40] Niesen F. H. Berglund H. Vedadi M. (2007). The use of differential scanning fluorimetry to detect ligand interactions that promote protein stability. Nat. Protoc. 2, 2212–2221. 10.1038/nprot.2007.321 17853878

[B41] Ortiz J. Artigas F. (1992). Effects of monoamine uptake inhibitors on extracellular and platelet 5-hydroxytryptamine in rat blood: different effects of clomipramine and fluoxetine. Br. J. Pharmacol. 105, 941–946. 10.1111/j.1476-5381.1992.tb09082.x 1387022 10.1111/j.1476-5381.1992.tb09082.x PMC1908726

[B42] Ouyang L. He G. Huang W. Song X. Wu F. Xiang M. (2012). Combined structure-based pharmacophore and 3D-QSAR studies on phenylalanine series compounds as TPH1 inhibitors. Int. J. Mol. Sci. 13, 5348–5363. 10.3390/ijms13055348 22754301 10.3390/ijms13055348 PMC3382768

[B43] Palucha-Poniewiera A. Podkowa K. Lenda T. Pilc A. (2017). The involvement of monoaminergic neurotransmission in the antidepressant-like action of scopolamine in the tail suspension test. Prog. Neuro-Psychopharmacol. Biol. Psychiatry 79, 155–161. 10.1016/j.pnpbp.2017.06.022 28647535

[B44] Petrassi M. Barber R. Be C. Beach S. Cox B. D'souza A. M. (2017). Identification of a novel allosteric inhibitory site on tryptophan hydroxylase 1 enabling unprecedented selectivity over all related hydroxylases. Front. Pharmacol. 8, 240 10.3389/fphar.2017.00240 28529483 PMC5418348

[B45] Petzer A. Pienaar A. Petzer J. P. (2013). The inhibition of monoamine oxidase by esomeprazole. Drug Res. 63, 462–467. 10.1055/s-0033-1345163 23677700

[B46] Pytka K. Dziubina A. Mlyniec K. Dziedziczak A. Zmudzka E. Furgala A. (2016a). The role of glutamatergic, GABA-ergic, and cholinergic receptors in depression and antidepressant-like effect. Pharmacol. Rep. 68, 443–450. 10.1016/j.pharep.2015.10.006 26922551

[B47] Pytka K. Podkowa K. Rapacz A. Podkowa A. Zmudzka E. Olczyk A. (2016b). The role of serotonergic, adrenergic and dopaminergic receptors in antidepressant-like effect. Pharmacol. Rep. 68, 263–274. 10.1016/j.pharep.2015.08.007 26922526

[B48] Sandler M. (1990). Monoamine oxidase inhibitors in depression: history and mythology. J. Psychopharmacol. 4, 136–139. 10.1177/026988119000400307 22282941

[B49] Slotkin T. A. (1999). Mary Bernheim and the discovery of monoamine oxidase. Brain Res. Bull. 50, 373 10.1016/s0361-9230(99)00110-0 10643441

[B50] Son S. Y. Ma J. Kondou Y. Yoshimura M. Yamashita E. Tsukihara T. (2008). Structure of human monoamine oxidase A at 2.2-A resolution: the control of opening the entry for substrates/inhibitors. Proc. Natl. Acad. Sci. U. S. A. 105, 5739–5744. 10.1073/pnas.0710626105 18391214 PMC2311356

[B51] Teigen K. Mckinney J. A. Haavik J. Martinez A. (2007). Selectivity and affinity determinants for ligand binding to the aromatic amino acid hydroxylases. Curr. Med. Chem. 14, 455–467. 10.2174/092986707779941023 17305546 10.2174/092986707779941023

[B52] Tong J. Meyer J. H. Furukawa Y. Boileau I. Chang L. J. Wilson A. A. (2013). Distribution of monoamine oxidase proteins in human brain: implications for brain imaging studies. J. Cerebr. Blood Flow Metabol. 33, 863–871. 10.1038/jcbfm.2013.19 PMC3677103 23403377

[B53] Tsamatsoulis M. Kapelios C. J. Charitos C. (2018). Hyperpyrexia in a patient with a left ventricular assist device: a diagnosis beyond the obvious. Interact. Cardiovasc. Thorac. Surg. 26, 883–884. 10.1093/icvts/ivx437 29346612

[B54] Waloen K. Kleppe R. Martinez A. Haavik J. (2017). Tyrosine and tryptophan hydroxylases as therapeutic targets in human disease. Expert Opin. Ther. Targets 21, 167–180. 10.1080/14728222.2017.1272581 27973928

[B55] Walther D. J. Bader M. (2003). A unique central tryptophan hydroxylase isoform. Biochem. Pharmacol. 66, 1673–1680. 10.1016/s0006-2952(03)00556-2 14563478

[B56] Walther D. J. Peter J. U. Bashammakh S. Hortnagl H. Voits M. Fink H. (2003). Synthesis of serotonin by a second tryptophan hydroxylase isoform. Science 299, 76 10.1126/science.1078197 12511643

[B57] Wang L. Erlandsen H. Haavik J. Knappskog P. M. Stevens R. C. (2002). Three-dimensional structure of human tryptophan hydroxylase and its implications for the biosynthesis of the neurotransmitters serotonin and melatonin. Biochemistry 41, 12569–12574. 10.1021/bi026561f 12379098

[B58] Wang Q. Timberlake M. A. II. Prall K. Dwivedi Y. (2017). The recent progress in animal models of depression. Prog. Neuro-Psychopharmacol. Biol. Psychiatry 77, 99–109. 10.1016/j.pnpbp.2017.04.008 PMC5605906 28396255

[B59] WHO (2019). WHO Model List of Essential Medicines [Online]. (Accessed June 2019).

[B60] Winge I. Mckinney J. A. Knappskog P. M. Haavik J. (2007). Characterization of wild-type and mutant forms of human tryptophan hydroxylase 2. J. Neurochem. 100, 1648–1657. 10.1111/j.1471-4159.2006.04290.x 17181551

[B61] Wu Q. Delamere N. A. (1997). Influence of bafilomycin A1 on pHi responses in cultured rabbit nonpigmented ciliary epithelium. Am. J. Physiol. 273, C1700–1706. 10.1152/ajpcell.1997.273.5.C1700 9374657

[B62] Zhang Z. Hamada H. Gerk P. M. (2019). Selectivity of dietary phenolics for inhibition of human monoamine oxidases A and B. BioMed Res. Int. 2019, 8361858 10.1155/2019/8361858 30809547 PMC6364133

[B63] Zimmer L. Luxen A. Giacomelli F. Pujol J. F. (2002). Short- and long-term effects of p-ethynylphenylalanine on brain serotonin levels. Neurochem. Res. 27, 269–275. 10.1023/A:1014998926763 11958527

