# Supplementary material for: Inhibition of Tryptophan Hydroxylases and Monoamine Oxidase-A by the Proton Pump Inhibitor, Omeprazole—In Vitro and In Vivo Investigations
Source: Front Pharmacol. 2020 Nov 26;11:593416. doi: 10.3389/fphar.2020.593416 (PMC7726444; doi:10.3389/fphar.2020.593416)
Supplement: Supplementary file 1 [file datasheet1.pdf]

Supplementary Material

Inhibition of tryptophan hydroxylases and monoamino oxidase-A by the proton pump inhibitor, omeprazole - *in vitro* and *in vivo* investigations

Nibal Betari[1]*†, Kristoffer Sahlholm[2,3,4]†, Xavier Morató[4,5,6], Héctor Godoy-Marín[4,5], Olga Jáuregui[7], Knut Teigen[1], Francisco Ciruela [4,5], Jan Haavik[1,8]

[1]Department of Biomedicine, University of Bergen, Bergen, Norway

[2]Department of Integrative Medical Biology, Wallenberg Centre for Molecular Medicine, Umeå University, Umeå, Sweden

[3]Department of Neuroscience, Karolinska Institutet, Stockholm, Sweden

[4]Pharmacology Unit, Department of Pathology and Experimental Therapeutics, Faculty of Medicine and Health Sciences, Institute of Neurosciences, University of Barcelona, L'Hospitalet de Llobregat, Spain.

[5]Neuropharmacology and Pain Group, Neuroscience Program, Institut d'Investigació Biomèdica de Bellvitge, IDIBELL, L'Hospitalet de Llobregat, Spain.

[6]Department of Clinical Neuroscience, Karolinska Institutet, Stockholm, Sweden

[7]Scientific and Technological Centers of University of Barcelona (CCiTUB), Barcelona, Spain.

[8] Bergen Center of Brain Plasticity, Division of Psychiatry, Haukeland University Hospital, Bergen, Norway

† These two authors contributed equally to the work

*** Correspondence:**

Nibal Betari

nibal.betari@uib.no

1.1 Supplementary Figures

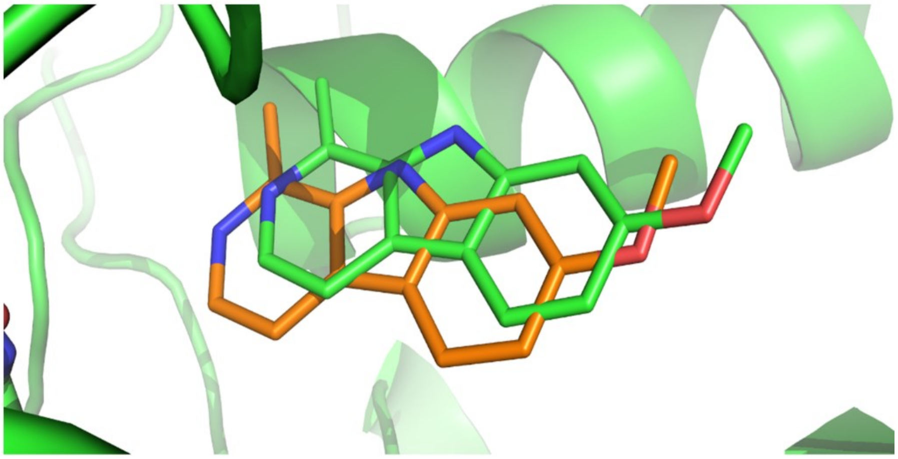

Supplementary Figure S1. Docking of harmine to MOA-A. Docking pose of harmine (green carbons) superimposed with the conformation of harmine from the x-ray structure (orange carbons). The main reason for the slight shift in position of harmine in the docked vs experimentally determined complex is likely due to the removal of water molecules prior to docking. Inclusion of water molecules resulted in docking poses even closer to the experimental binding pose. However, water molecules were removed prior to the docking procedure to better explore the binding pocket of the receptors.

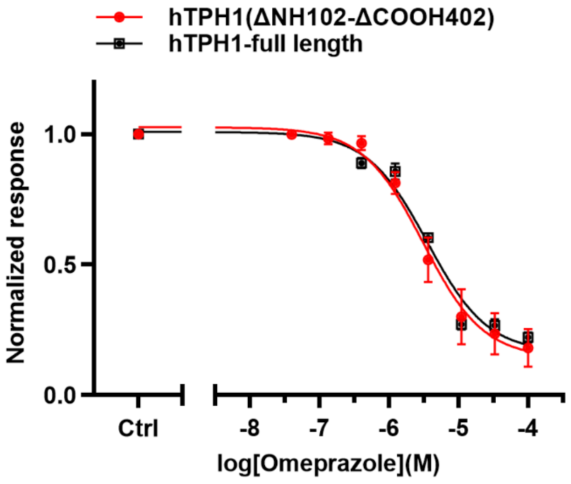

Supplementary Figure S2. Comparing the potency of omeprazole in inhibiting full-length and doubly truncated tryptophan hydroxylase 1. Omeprazole demonstrated similar inhibitory activity at full-length TPH1 IC_{50} = 3.55 (95% confidence interval; 2.31 to 5.49) μM and at doubly truncated hTPH1(ΔNH102-ΔCOOH402) IC_{50} = 3.09 (95% confidence interval; 2.53 to 3.77) μM.

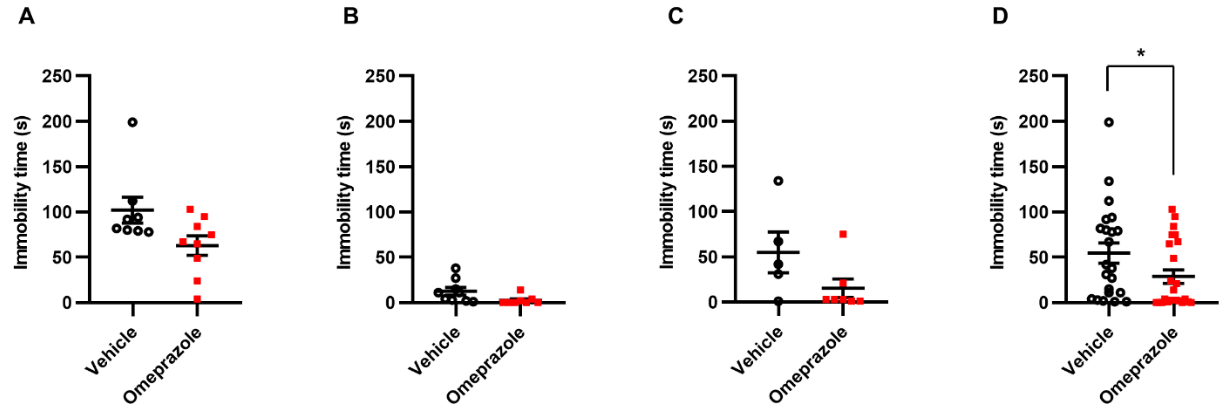

Supplementary Figure 3. In vivo effects of 100 mg/kg omeprazole on immobility time in the tail suspension test. Immobility time in the tail suspension test, performed on three different groups of animals. Statistical significance was evaluated using the Mann-Whitney test; A) Group 1, $p = 0.059$ B) Group 2; $p = 0.014$ C) Group3; $p = 0.193$ D) Pooled data from all three groups; *, $p = 0.028$.
